# A dataset of tree heights in mangrove and non-mangrove trees in Malaysia derived from multiple measurement methods

**DOI:** 10.1016/j.dib.2020.106386

**Published:** 2020-10-09

**Authors:** Ibrahim Sunkanmi Saliu, Giovanna Wolswijk, Behara Satyanarayana, Muhammad Amir Bin Fisol, Charles Decannière, Richard Lucas, Viviana Otero, Farid Dahdouh-Guebas

**Affiliations:** a Systems Ecology and Resource Management Research Unit, Department of Organism Biology, Faculty of Sciences, Université Libre de Bruxelles (ULB), Avenue F.D. Roosevelt 50, CPi 264/1, B-1050 Brussels, Belgium; b Mangrove Research Unit (MARU), Institute of Oceanography and Environment (INOS), Universiti Malaysia Terengganu (UMT), 21030 Kuala Nerus, Terengganu, Malaysia; c Landscape ecology and Vegetal Production Systems Research Unit / Agroecology Lab, Inter-Faculty School of Bioengineers, Université Libre de Bruxelles (ULB) Avenue F.D. Roosevelt 50, CPi 264/1, B-1050 Brussels, Belgium; d Centre for Ecosystem Science, School of Biological, Earth and Environmental Sciences, University of New South, Wales (UNSW), Australia; e Earth Observation and Ecosystems Dynamics Laboratory, Aberystwyth University, Penglais, Aberystwyth SY23 3FL, UK; f Ecology & Biodiversity / General Botany and Nature Management Research Unit, Department of Biology, Faculty of Sciences and Bio-engineering Sciences, Vrije Universiteit Brussel (VUB), VUB-APNA-WE Pleinlaan 2, B-1050 Brussels, Belgium

**Keywords:** Tree height, Height-diameter allometry, Hypsometer, UAV

## Abstract

The dataset contains tree height data collected in 200 mangrove and non-mangrove trees sampled in various sites in Malaysia. Different height measurement methods were performed, including visual measurements (stick, thumb rule) and precision field instruments (clinometer, laser rangefinder and altimeter), which were compared against benchmark values obtained using an unmanned aerial vehicle (UAV) and a Leica distometer. The core data have been analysed and interpreted in the paper by Saliu *et al.* ‘’An accuracy analysis of mangrove tree height mensuration using forestry techniques, hypsometers and UAVs ’’ [1], in which the accuracy of each method for tree height measurement was discussed.

## Specifications Table

**Table:** 

Subject	Forestry
Specific subject area	Tree height measurements
Type of data	Table
How data were acquired	Thumb rule, Stick method, Suunto PM - 5/360 PC clinometer, Nikon 550 Forestry Pro Laser Rangefinder, Blume - Leiss BL 60 Altimeter, UAV (drone) DJI Phantom 3, Leica distometer D2 Bluetooth (Leica Geosystems), GPSMAP 64s global positioning system (GPS) (Garmin limited), diameter tape.
Data format	Raw
Parameters for data collection	Each tree height measurement was derived from various techniques, *i.e.* thumb rule, stick method, Suunto clinometer, Nikon 550 Forestry Pro laser rangefinder and Blume Leiss BL 60 altimeter. Control heights were obtained through an Unmanned Aerial Vehicle (UAV – DJI Phantom 3 Professional) and Leica distometer.
Description of data collection	Individual trees were targeted to measure their height with different equipment. Targeted trees were either vertically straight (with the top vertically above the base) or with a slight lean (exhibiting not more than 5° inclination from the perpendicular).
Data source location	Universiti Malaysia Terengganu (UMT) campus Kuala Nerus, Terengganu, Malaysia GPS coordinates: 05° 24.52’ N; 103° 05.33’ E Matang Mangrove Forest Reserve Kuala Sepetang, Perak, Malaysia GPS coordinates: 04°15’ – 05°01’N; 100°02’ – 100°45’E Cafeteria near to the forest department Kuala Sepetang 04ᵒ 50.59’ N; 100ᵒ 38.00’ E Compartment 19A 04ᵒ 50.98’ N; 100ᵒ 38.83’ E
Data accessibility	With the article
Related research article	I.S. Saliu, B. Satyanarayana, M.A. Fisol, G. Wolswijk, C. Decannière, R. Lucas, V. Otero, F. Dahdouh-Guebas (2020), An accuracy analysis of mangrove tree height mensuration using forestry techniques, hypsometers and UAVs, *Estuarine, Coastal and Shelf Science* 106971*. https://doi.org/10.1016/j.ecss.2020.106971* (In press).

## Value of the Data

• Due to limited scientific investigations on this subject, the present data provides a valuable information on mangrove and non-mangrove tree height measurements obtained from different forest inventory techniques.
• The data can be used to develop tree height-diameter allometry in mangrove and non-mangrove species in Malaysia, which can be used for further forest inventory applications, considering the difficulty of tree height measurements in mangrove species.
• The present dataset comprises 200 tree height measurements considered to be beneficial to other tree height studies in Malaysia for statistical validation, accuracy assessment and forest biomass derivations.
• The outcomes of this study would be able to help the researchers elsewhere to acquire most reliable tree heights by selecting appropriate tools, along with less labour and time saving benefits.

## 1 Data Description

The data reported in Table 1, that was analysed and discussed in the paper by Saliu *et al*. [1] (173 out of 200 trees were used for the analysis due to some missing data), represents a description of the sampling locations where the individual tree height measurements were obtained through different forest inventory techniques *i.e.,* thumb rule, stick method, clinometer, laser rangefinder and altimeter. Control heights were obtained with the help of an Unmanned Aerial Vehicle (UAV – DJI Phantom 3 Professional) and a distometer. Table 1 also reports the values of stem diameter (D_130_) and inclination angle of each individual tree. Altogether 200 trees were considered of which 146 represent non-mangrove trees on the Universiti Malaysia Terengganu (UMT) campus (State Terengganu) and the rest (54) mangrove trees at the Matang Mangrove Forest Reserve (MMFR) (State Perak) in Peninsular Malaysia. At the UMT, tree species chosen were *Archontophoenix alexandrae* (F. Muell.) H. Wendl. & Drude, *Millettia pinnata* (L.) Panigrahi, *Casuarina equisetifolia* L., *Terminalia ivorensis* A. Chev.*, Polyalthia longifolia* Sonn.*, Syzygium polyanthum* Wight*, Mangifera indica* L*. and Picea* spp*.* In the case of MMFR, the sampled trees near the cafeteria (Kuala Sepetang Forestry Department) included *Casuarina equisetifolia, Avicennia* spp*., Rhizophora* spp*.* and *Archontophoenix alexandrae*, whereas in the compartment no. 19A (managed mangrove stand) it was all *Rhizophora apiculata* Bl. or *R. mucronata* Lamk*.* It is also noteworthy that the trees being sampled in the UMT campus and at the MMFR cafeteria were largely isolated individuals, while in the compartment 19A the trees were located adjacent to a closed canopy.

**Table 1:** Sampling locations for each individual tree and tree height measurements with different methods. UMT: Universiti Malaysia Terengganu campus, MMFR: Matang Mangrove Forest Reserve (with indication of exact location as Cafeteria or Compartment 19A); genus names: *A.: Archontophoenix, T.: Terminalia, P.: Polyalthia, Ml.: Millettia, S.: Syzygium, C.: Casuarina, Mn.: Mangifera*; D_130_: diameter measure at 130 cm from the ground and along the stem; Incl.: inclination; tree height measurements methods: TR: thumb rule, SM: stick method, C: clinometer, LR: laser rangefinder, A: altimeter, UAV: Unmanned Aerial Vehicle, D: Leica distometer (control height); N.A.: Not available values (cases in which tree species was not identified or measurement of tree height in certain areas was not possible with certain methods, in the latter case these trees were not included in the analysis by Saliu *et al*. [1]).

Tree description					Tree height (m)						
N°	Location	Species	D_130_ (cm)	Incl. (°)	TR	SM	C	LR	A	UAV	D
1	UMT	N.A.	20.70	0.00	4.85	4.33	6.04	5.20	6.00	6.40	6.80
2	UMT	N.A.	18.20	3.00	5.80	6.46	6.76	6.20	6.50	7.00	7.60
3	UMT	N.A.	16.50	0.00	4.30	4.58	4.52	4.00	4.30	4.80	4.80
4	UMT	N.A.	16.50	0.00	6.31	6.32	5.76	5.60	5.80	6.70	6.50
5	UMT	N.A.	16.60	0.00	5.49	6.47	6.04	5.40	5.90	6.10	6.20
6	UMT	N.A.	19.30	1.00	6.53	6.40	7.06	6.80	7.30	7.90	7.70
7	UMT	N.A.	16.80	3.00	4.50	6.03	5.90	5.80	5.90	5.90	6.90
8	UMT	*A. alexandrae*	28.30	0.00	9.07	10.31	9.58	8.60	8.60	9.70	10.00
9	UMT	*A. alexandrae*	25.90	0.00	7.32	8.57	7.81	7.40	7.10	7.60	8.00
10	UMT	*A. alexandrae*	23.80	0.00	5.30	6.79	5.90	5.40	5.40	5.50	5.60
11	UMT	*A. alexandrae*	28.80	0.00	6.10	6.72	6.19	6.00	6.10	5.50	5.60
12	UMT	*A. alexandrae*	26.60	0.00	9.60	12.44	9.91	9.40	9.50	10.00	9.70
13	UMT	N.A.	30.90	0.00	9.10	9.77	9.41	8.20	8.50	8.70	9.60
14	UMT	*T. ivorensis*	35.40	1.00	6.20	7.02	9.68	8.20	8.50	8.60	9.10
15	UMT	*T. ivorensis*	26.00	4.00	3.90	3.97	6.62	6.40	7.10	7.00	7.40
16	UMT	*T. ivorensis*	17.40	0.00	4.90	6.17	5.76	4.60	4.90	5.20	5.60
17	UMT	*T. ivorensis*	16.50	2.00	9.03	4.46	6.47	5.40	5.80	5.90	5.80
18	UMT	*T. ivorensis*	17.00	0.00	5.78	6.65	5.62	4.40	4.60	4.50	4.60
19	UMT	*T. ivorensis*	14.90	2.00	4.12	4.17	7.36	6.00	6.60	5.90	6.10
20	UMT	*T. ivorensis*	10.80	1.00	4.73	5.73	5.62	4.80	5.40	5.10	5.20
21	UMT	N.A.	27.40	0.00	11.24	11.95	10.61	10.20	10.10	10.40	10.40
22	UMT	N.A.	23.70	0.00	9.86	10.85	10.26	9.40	9.50	10.50	10.20
23	UMT	*P. longifolia*	23.40	0.00	9.10	10.92	10.61	9.80	10.00	10.40	10.20
24	UMT	*P. longifolia*	32.80	2.00	10.32	14.59	12.10	12.20	11.90	13.80	12.80
25	UMT	*P. longifolia*	23.50	0.00	8.21	11.26	9.91	9.80	9.70	12.50	10.50
26	UMT	*Ml. pinnata*	27.60	3.00	6.77	9.63	9.74	7.60	9.00	8.50	8.70
27	UMT	N.A.	21.80	3.00	7.26	8.98	10.09	8.40	8.70	8.80	9.00
28	UMT	N.A.	17.70	4.00	6.56	8.85	9.58	8.20	8.50	9.10	9.60
29	UMT	N.A.	25.40	2.00	6.86	9.60	9.74	7.80	8.50	8.40	8.90
30	UMT	N.A.	32.60	3.00	6.84	9.73	9.91	7.00	9.50	5.20	6.40
31	UMT	N.A.	18.30	2.00	6.91	11.63	12.10	9.00	11.30	9.50	10.60
32	UMT	N.A.	32.50	5.00	7.27	10.49	10.09	8.80	9.80	9.20	9.30
33	UMT	N.A.	24.90	2.00	6.42	9.39	9.58	8.00	9.20	8.90	8.50
34	UMT	N.A.	22.80	1.00	6.25	8.75	8.44	7.20	8.10	7.30	7.90
35	UMT	N.A.	25.90	3.00	6.85	10.62	10.44	8.20	10.10	8.50	9.10
36	UMT	*A. alexandrae*	18.20	0.00	9.72	11.97	11.64	10.80	10.80	11.30	11.50
37	UMT	*A. alexandrae*	20.00	0.00	8.95	12.14	10.26	10.40	10.00	10.40	10.40
38	UMT	*A. alexandrae*	19.00	0.00	9.35	11.80	9.64	9.80	9.80	10.90	10.40
39	UMT	*A. alexandrae*	19.80	2.00	9.37	12.00	11.06	10.60	10.40	10.80	11.00
40	UMT	N.A.	30.70	3.00	7.10	10.12	9.08	8.40	8.90	8.40	8.60
41	UMT	N.A.	18.40	3.00	5.17	7.14	6.33	6.00	6.10	5.80	6.00
42	UMT	N.A.	34.70	0.00	5.14	6.40	5.62	5.80	5.80	7.30	6.20
43	UMT	N.A.	26.80	2.00	6.70	9.14	8.12	8.00	8.40	8.30	8.30
44	UMT	N.A.	30.40	2.00	10.96	15.82	13.32	12.60	12.40	12.70	12.80
45	UMT	N.A.	39.80	1.00	11.80	16.78	9.08	7.40	8.60	9.30	9.10
46	UMT	N.A.	43.30	1.00	7.10	8.44	15.60	11.90	14.30	11.40	11.70
47	UMT	N.A.	42.50	2.00	11.65	17.07	15.35	13.80	14.50	14.10	14.60
48	UMT	N.A.	42.80	2.00	12.61	16.92	18.68	15.00	16.80	15.00	15.40
49	UMT	N.A.	46.00	4.00	13.95	18.97	15.83	13.40	14.80	13.80	14.20
50	UMT	N.A.	41.60	0.00	5.39	10.03	7.81	7.40	8.70	10.20	10.70
51	UMT	N.A.	40.30	0.00	9.40	9.80	8.92	8.80	9.00	9.00	9.50
52	UMT	N.A.	45.70	4.00	9.50	10.18	10.61	9.80	10.00	10.70	10.90
53	UMT	N.A.	44.10	3.00	12.36	17.67	14.10	14.40	10.90	13.90	14.20
54	UMT	N.A.	45.80	2.00	8.46	9.69	10.26	9.40	9.90	9.90	10.30
55	UMT	N.A.	36.60	0.00	6.78	9.13	8.75	9.80	8.60	9.70	10.20
56	UMT	N.A.	31.80	3.00	7.74	9.68	8.75	8.80	8.70	9.30	9.80
57	UMT	N.A.	43.70	4.00	6.93	8.88	10.79	10.40	10.70	10.20	10.80
58	UMT	N.A.	34.40	1.00	9.05	11.05	9.58	9.20	9.70	9.90	10.40
59	UMT	N.A.	46.50	3.00	9.88	11.35	10.26	10.30	10.30	10.80	11.60
60	UMT	N.A.	49.10	0.00	15.10	21.20	17.55	17.00	17.70	18.90	19.10
61	UMT	N.A.	44.70	0.00	13.46	22.56	18.92	17.90	18.30	19.60	19.60
62	UMT	N.A.	35.30	1.00	11.70	21.03	16.56	16.00	16.90	17.50	17.20
63	UMT	N.A.	56.80	2.00	10.64	15.16	12.82	12.00	12.00	11.70	12.30
64	UMT	N.A.	52.20	0.00	10.40	15.10	13.12	13.20	13.10	12.10	12.60
65	UMT	N.A.	50.10	1.00	12.20	21.61	15.59	16.00	16.10	15.00	15.90
66	UMT	N.A.	36.70	0.00	12.59	19.31	16.56	16.20	16.90	16.70	17.20
67	UMT	N.A.	42.20	1.00	12.75	15.51	13.42	13.60	13.90	13.00	13.80
68	UMT	N.A.	43.80	1.00	7.72	11.33	10.50	9.60	10.00	9.80	10.20
69	UMT	N.A.	44.00	0.00	10.76	20.06	16.56	16.00	16.60	15.80	16.30
70	UMT	N.A.	48.60	0.00	12.34	18.26	14.64	14.00	14.40	13.90	14.30
71	UMT	N.A.	45.00	3.00	12.29	18.69	16.23	16.20	16.50	16.20	17.00
72	UMT	N.A.	54.00	1.00	11.32	17.77	15.59	14.70	15.10	14.50	14.80
73	UMT	N.A.	28.40	2.00	6.04	5.78	8.12	7.20	8.30	7.20	7.50
74	UMT	N.A.	28.70	1.00	8.15	8.46	6.76	6.40	6.80	7.50	7.50
75	UMT	N.A.	19.80	3.00	7.94	6.22	6.76	6.20	6.40	5.90	5.90
76	UMT	N.A.	27.10	0.00	7.02	6.20	10.09	8.80	9.70	8.90	9.00
77	UMT	N.A.	26.30	2.00	9.21	10.82	8.12	7.20	7.70	7.30	7.20
78	UMT	N.A.	19.00	0.00	8.60	7.99	7.81	6.80	7.70	7.40	7.10
79	UMT	N.A.	25.00	3.00	11.32	6.99	12.70	10.20	12.40	10.20	10.40
80	UMT	N.A.	21.10	1.00	6.66	8.16	8.12	6.60	7.10	7.70	7.70
81	UMT	N.A.	19.20	1.00	8.47	18.16	9.24	8.60	8.60	9.60	9.70
82	UMT	N.A.	24.40	0.00	7.86	10.37	8.44	7.60	8.10	7.80	8.10
83	UMT	N.A.	18.60	0.00	6.38	9.07	7.36	6.80	7.10	6.30	6.80
84	UMT	N.A.	25.50	0.00	9.15	10.60	9.41	7.60	9.00	8.00	8.20
85	UMT	N.A.	20.20	1.00	8.47	10.41	8.28	7.00	8.10	7.10	7.60
86	UMT	N.A.	14.20	0.00	7.61	10.14	7.97	6.80	8.00	7.20	7.30
87	UMT	N.A.	19.00	1.00	8.83	9.59	9.08	8.60	9.10	8.10	8.70
88	UMT	N.A.	18.10	1.00	7.63	9.47	8.28	7.60	8.10	8.40	8.50
89	UMT	N.A.	33.20	0.00	7.28	9.19	7.81	7.80	7.80	7.40	7.70
90	UMT	N.A.	39.10	0.00	7.32	8.34	7.21	7.40	7.20	7.40	7.30
91	UMT	N.A.	28.00	0.00	11.60	14.66	11.53	12.80	11.70	12.90	13.20
92	UMT	N.A.	42.40	0.00	9.52	10.43	8.92	9.40	9.00	9.60	9.90
93	UMT	N.A.	47.30	0.00	8.00	12.90	12.69	12.60	12.40	13.00	13.20
94	UMT	N.A.	21.70	0.00	12.15	13.24	12.46	12.70	12.10	13.00	13.30
95	UMT	N.A.	48.10	1.00	11.05	13.58	12.46	11.20	11.80	12.30	12.40
96	UMT	N.A.	37.20	1.00	16.20	17.90	17.23	15.60	16.50	16.30	16.50
97	UMT	N.A.	39.50	0.00	20.30	14.37	14.64	11.30	12.90	13.90	14.20
98	UMT	N.A.	34.10	0.00	20.10	14.79	14.64	13.40	13.10	13.00	13.30
99	UMT	N.A.	23.20	0.00	10.09	10.79	10.77	10.30	10.00	10.60	10.80
100	UMT	N.A.	23.60	0.00	8.28	9.34	9.64	8.40	8.00	8.60	9.00
101	UMT	N.A.	25.30	0.00	9.60	12.14	11.93	11.60	11.20	11.10	11.30
102	UMT	*A. alexandrae*	23.90	0.00	4.75	5.29	5.62	5.20	5.20	5.20	5.30
103	UMT	*A. alexandrae*	28.90	0.00	6.32	6.32	6.33	5.80	5.60	6.70	6.70
104	UMT	*A. alexandrae*	33.40	0.00	9.20	9.06	8.12	7.80	7.80	7.90	8.20
105	UMT	*T. ivorensis*	34.10	0.00	6.28	7.87	6.96	6.50	6.50	7.40	7.60
106	UMT	*A. alexandrae*	31.70	0.00	6.78	8.83	7.91	7.20	7.00	7.80	7.60
107	UMT	*A. alexandrae*	32.50	0.00	6.43	8.64	7.98	8.00	7.10	8.70	8.80
108	UMT	N.A.	51.80	1.00	20.75	18.64	22.61	23.20	22.00	22.00	22.70
109	UMT	N.A.	25.50	0.00	24.30	26.43	21.84	20.40	21.10	21.80	23.00
110	UMT	N.A.	52.70	4.00	15.95	21.01	18.23	17.60	17.90	18.10	18.10
111	UMT	*S. polyanthum*	18.20	0.00	4.20	5.50	4.93	4.60	4.50	5.50	5.70
112	UMT	A. alexandrae	18.30	0.00	5.00	5.76	5.20	4.90	4.90	5.60	5.70
113	UMT	*A. alexandrae*	17.90	0.00	4.91	5.40	4.93	4.90	5.00	5.50	5.60
114	UMT	*A. alexandrae*	20.20	0.00	5.20	5.92	5.34	5.20	5.30	5.80	5.90
115	UMT	*A. alexandrae*	20.60	2.00	4.95	4.95	4.79	4.40	4.30	5.50	5.40
116	UMT	*A. alexandrae*	24.90	1.00	9.10	11.66	9.28	9.60	9.40	11.90	11.50
117	UMT	*A. alexandrae*	18.10	0.00	4.44	4.50	4.50	4.20	4.40	4.45	4.45
118	UMT	*A. alexandrae*	44.90	1.00	7.84	8.18	7.06	7.00	7.80	6.90	7.60
119	UMT	*Picea* spp*.*	20.40	0.00	6.53	6.70	6.47	6.20	6.50	6.60	6.90
120	UMT	*A. alexandrae*	16.50	0.00	4.91	5.44	5.20	4.80	4.90	5.10	5.40
121	UMT	*Ml. pinnata*	47.70	1.00	7.94	10.42	9.92	9.60	9.50	9.70	10.00
122	UMT	*Mn. indica*	39.50	1.00	9.87	12.15	11.93	10.80	11.30	7.80	8.20
123	UMT	*Ml. pinnata*	25.50	5.00	9.33	14.47	10.49	10.40	9.70	9.20	10.70
124	UMT	*Ml. pinnata*	58.60	1.00	20.02	28.24	21.98	20.60	20.30	24.10	24.60
125	UMT	*C. equisetifolia*	23.20	1.00	9.00	8.14	7.33	7.60	7.10	7.80	8.00
126	UMT	N.A.	11.50	0.00	5.13	4.80	4.52	4.40	4.30	5.00	5.10
127	UMT	N.A.	21.30	0.00	7.79	10.42	8.59	9.00	8.50	9.20	9.40
128	UMT	*A. alexandrae*	24.80	0.00	9.77	11.48	10.93	11.40	11.00	11.90	12.20
129	UMT	*A. alexandrae*	36.00	0.00	7.52	7.68	7.52	7.40	7.30	7.40	7.80
130	UMT	*A. alexandrae*	17.20	0.00	8.95	10.25	9.58	9.20	9.00	8.20	8.80
131	UMT	*A. alexandrae*	27.10	0.00	8.62	12.21	10.71	11.00	10.50	10.90	11.20
132	UMT	*A. alexandrae*	21.60	0.00	7.50	9.45	8.88	8.20	8.00	8.00	8.20
133	UMT	*A. alexandrae*	33.70	0.00	12.28	14.38	12.23	11.80	11.40	12.00	12.20
134	UMT	*A. alexandrae*	25.80	0.00	14.80	15.21	12.23	12.40	12.00	13.10	13.20
135	UMT	*A. alexandrae*	41.40	0.00	9.00	10.74	10.09	10.00	9.70	11.50	11.60
136	UMT	*A. alexandrae*	25.80	0.00	8.31	7.76	7.06	6.80	6.90	7.30	7.50
137	UMT	*A. alexandrae*	22.90	0.00	10.26	9.38	10.09	10.10	9.60	9.80	9.70
138	UMT	*A. alexandrae*	26.70	0.00	13.62	17.77	14.96	15.20	14.90	15.10	15.30
139	UMT	*A. alexandrae*	32.50	0.00	13.79	17.71	15.27	14.90	14.50	14.60	14.90
140	UMT	*A. alexandrae*	25.50	0.00	7.51	8.04	7.97	8.00	7.60	7.20	7.70
141	UMT	*A. alexandrae*	32.10	0.00	11.50	12.92	11.57	12.20	11.00	10.20	10.50
142	UMT	*A. alexandrae*	22.90	0.00	10.39	12.38	10.71	11.40	10.60	10.20	10.30
143	UMT	*A. alexandrae*	31.20	0.00	8.03	12.69	10.93	11.00	10.00	11.80	12.20
144	UMT	*A. alexandrae*	34.70	0.00	10.78	12.53	12.69	11.60	11.00	12.10	13.90
145	UMT	*A. alexandrae*	29.60	0.00	15.46	18.26	15.09	15.40	14.70	13.30	14.70
146	UMT	*A. alexandrae*	13.70	0.00	5.00	6.77	5.48	5.60	5.60	5.60	5.70
147	MMFR Cafeteria	*C. equisetifolia*	45.50	5.00	25.11	33.00	29.61	25.40	27.60	23.70	23.70
148	MMFR Cafeteria	*C. equisetifolia*	27.10	5.00	16.09	18.69	16.23	15.80	16.30	15.70	15.60
149	MMFR Cafeteria	*C. equisetifolia*	14.50	3.00	17.40	18.35	16.56	16.80	15.90	16.00	16.90
150	MMFR Cafeteria	*Avicennia* spp*.*	24.60	0.00	21.00	20.90	18.58	18.00	18.60	18.50	17.90
151	MMFR Cafeteria	*Rhizophora* spp*.*	12.20	0.00	8.28	7.59	7.71	7.20	7.70	7.20	7.40
152	MMFR Cafeteria	N.A.	17.40	0.00	14.40	14.04	16.60	14.60	16.00	14.60	14.40
153	MMFR Cafeteria	N.A.	26.80	4.00	8.36	9.26	8.80	8.40	8.50	8.20	8.50
154	MMFR Cafeteria	N.A.	31.90	0.00	12.00	14.28	12.82	10.50	11.90	11.10	10.90
155	MMFR Cafeteria	N.A.	23.40	3.00	8.72	10.38	9.92	9.20	9.50	9.00	9.10
156	MMFR Cafeteria	*A. alexandrae*	22.90	0.00	11.00	11.57	11.94	11.40	11.20	11.00	11.30
157	MMFR Cafeteria	N.A.	18.50	2.00	7.55	10.14	9.58	7.40	9.10	7.80	7.70
158	MMFR Cafeteria	N.A.	19.50	2.00	6.94	8.47	8.59	7.40	8.10	8.20	8.00
159	MMFR Cafeteria	N.A.	19.50	1.00	7.56	7.24	8.12	8.00	8.10	7.90	8.10
160	MMFR Cafeteria	N.A.	26.80	2.00	5.98	6.63	6.76	6.30	6.50	6.50	6.60
161	MMFR Cafeteria	N.A.	15.60	0.00	5.33	6.95	6.76	6.00	6.60	6.20	6.30
162	MMFR Cafeteria	N.A.	12.20	0.00	12.80	12.80	12.01	12.40	12.40	12.00	12.30
163	MMFR Cafeteria	N.A.	16.40	4.00	10.40	10.22	10.09	9.40	10.20	10.10	9.80
164	MMFR Cafeteria	*C. equisetifolia*	19.30	2.00	7.59	8.91	8.92	9.00	8.70	9.20	9.10
165	MMFR Cafeteria	*C. equisetifolia*	36.40	3.00	13.40	10.77	10.77	10.90	10.70	14.00	13.50
166	MMFR Cafeteria	*C. equisetifolia*	23.10	0.00	16.00	14.74	15.59	16.20	15.40	16.80	16.50
167	MMFR Cafeteria	*C. equisetifolia*	19.50	3.00	17.40	15.90	17.55	18.40	17.80	19.50	19.10
168	MMFR Cafeteria	*Rhizophora* spp*.*	26.70	0.00	14.00	16.50	14.33	12.60	13.30	15.60	14.10
169	MMFR Cafeteria	*Rhizophora* spp.	19.30	2.00	15.80	20.59	17.22	14.60	16.20	16.10	15.80
170	MMFR Cafeteria	*Rhizophora* spp*.*	37.60	5.00	13.80	15.98	14.03	12.80	14.10	13.50	13.00
171	MMFR Cafeteria	N.A.	26.30	2.00	11.40	11.60	10.93	10.00	11.00	11.50	11.20
172	MMFR Cafeteria	N.A.	19.10	2.00	9.80	11.96	10.49	10.80	10.60	11.20	11.10
173	MMFR Cafeteria	N.A.	21.50	1.00	10.40	15.40	14.33	13.80	14.10	13.10	13.60
174	MMFR 19A	*Rhizophora* spp*.*	21.70	0.00	N.A.	18.25	25.04	22.20	21.90	21.90	N.A.
175	MMFR 19A	*Rhizophora* spp*.*	21.80	0.00	N.A.	17.63	24.21	22.40	22.00	22.00	N.A.
176	MMFR 19A	*Rhizophora* spp*.*	17.00	0.00	N.A.	18.31	24.61	22.40	22.80	22.20	N.A.
177	MMFR 19A	*Rhizophora* spp*.*	25.80	1.00	N.A.	19.44	24.61	22.20	21.90	21.80	N.A.
178	MMFR 19A	*Rhizophora* spp*.*	16.70	0.00	N.A.	17.13	21.25	19.40	19.80	20.40	N.A.
179	MMFR 19A	*Rhizophora* spp*.*	16.10	0.00	N.A.	17.04	19.16	18.40	17.00	18.20	N.A.
180	MMFR 19A	*Rhizophora* spp*.*	14.80	0.00	N.A.	14.57	17.51	18.00	17.50	17.30	N.A.
181	MMFR 19A	*Rhizophora* spp*.*	16.50	0.00	N.A.	15.77	15.35	17.00	16.50	17.20	N.A.
182	MMFR 19A	*Rhizophora* spp*.*	15.40	0.00	N.A.	12.80	17.89	15.40	16.70	16.00	N.A.
183	MMFR 19A	*Rhizophora* spp*.*	17.50	0.00	N.A.	8.90	9.74	9.20	8.90	9.80	N.A.
184	MMFR 19A	*Rhizophora* spp*.*	13.50	0.00	N.A.	18.10	23.02	18.80	21.40	19.30	N.A.
185	MMFR 19A	*Rhizophora* spp*.*	16.70	0.00	N.A.	16.96	15.09	16.40	14.80	N.A.	N.A.
186	MMFR 19A	*Rhizophora* spp*.*	17.40	0.00	N.A.	15.14	16.67	16.80	16.30	16.40	N.A.
187	MMFR 19A	*Rhizophora* spp*.*	25.50	N.A.	N.A.	12.37	14.59	16.00	14.60	14.40	N.A.
188	MMFR 19A	*Rhizophora* spp*.*	25.00	N.A.	N.A.	17.60	22.68	20.40	22.10	N.A.	N.A.
189	MMFR 19A	*Rhizophora* spp*.*	22.30	N.A.	N.A.	13.23	19.93	17.60	14.50	N.A.	N.A.
190	MMFR 19A	*Rhizophora* spp*.*	16.10	N.A.	N.A.	17.55	19.93	21.00	18.90	19.00	N.A.
191	MMFR 19A	*Rhizophora* spp*.*	24.10	N.A.	N.A.	23.65	29.09	28.60	26.00	27.25	N.A.
192	MMFR 19A	*Rhizophora* spp*.*	30.50	N.A.	N.A.	23.21	27.68	24.80	25.00	26.67	N.A.
193	MMFR 19A	*Rhizophora* spp*.*	27.00	N.A.	N.A.	23.66	28.14	27.00	25.90	29.08	N.A.
194	MMFR 19A	*Rhizophora* spp*.*	25.00	N.A.	N.A.	11.97	15.16	14.30	13.80	14.20	N.A.
195	MMFR 19A	*Rhizophora* spp*.*	23.60	N.A.	N.A.	15.00	17.94	17.94	16.54	15.90	N.A.
196	MMFR 19A	*Rhizophora* spp*.*	24.60	N.A.	N.A.	10.68	14.35	13.05	13.25	12.20	N.A.
197	MMFR 19A	*Rhizophora* spp*.*	26.70	N.A.	N.A.	16.74	22.45	22.10	21.20	21.00	N.A.
198	MMFR 19A	*Rhizophora* spp*.*	21.80	N.A.	N.A.	23.45	28.30	28.13	26.93	27.20	N.A.
199	MMFR 19A	*Rhizophora* spp*.*	25.90	N.A.	N.A.	26.74	29.45	30.37	28.27	27.40	N.A.
200	MMFR 19A	*Rhizophora* spp*.*	18.70	N.A.	N.A.	24.39	29.84	28.90	28.30	25.10	N.A.

## 2 Experimental Design, Materials and Methods

Tree height measurements in the UMT and MMFR sites were acquired through different forest inventory techniques *i.e.*, thumb rule, stick method, Suunto PM - 5/360 PC clinometer (Finland), Nikon 550 Forestry Pro laser rangefinder (Republic of Ireland) , and Blume Leiss BL 60 altimeter(Germany), along with an Unmanned Aerial vehicle (UAV – DJI Phantom 3 Professional, China) and a Leica Geosystems Distometer D2 Bluetooth (Switzerland), the latter considered as control measurements. All trees were randomly selected and marked (with permanent marker) for cross checking, if necessary.

**Fig. 1:**
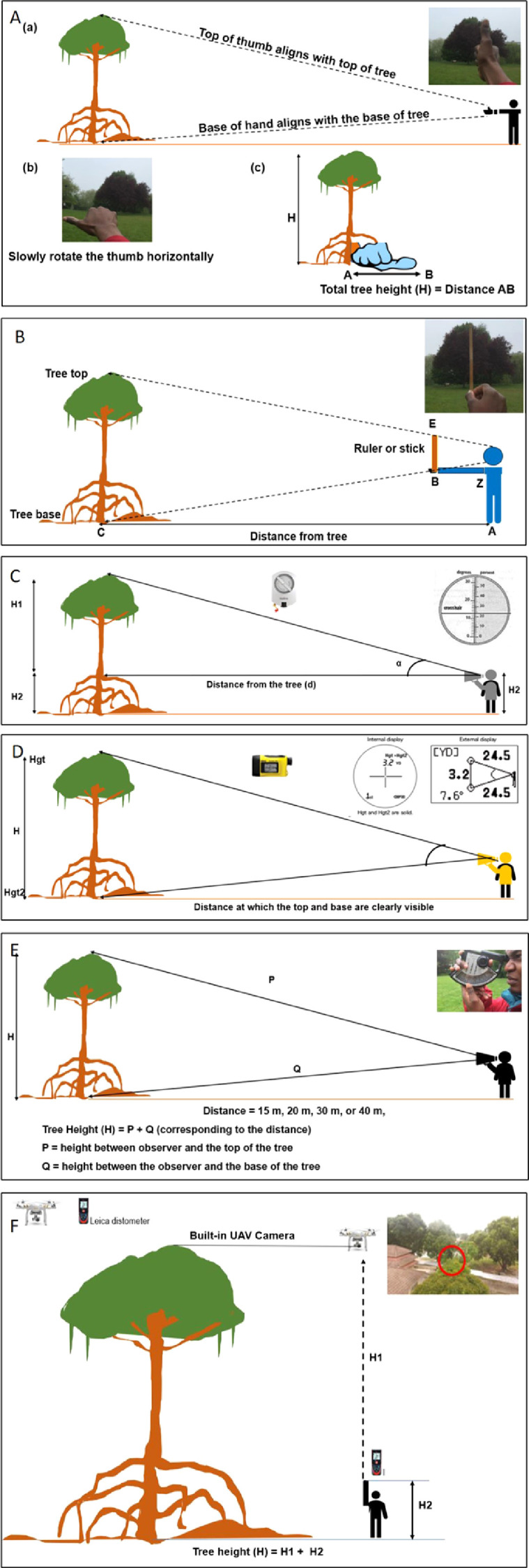
Description of different tree height measurement methods used. A) thumb rule method (Distance AB = Height of the tree). B) Stick method. C) Clinometer, the internal view of the device is shown with the degree and the percentage scales. D) Laser rangefinder. An observer shooting device to two points; Hgt and Hgt2. Internal and external displays showing results from measurement between two points (adopted from https://www.nhbs.com/nikon-forestry-pro-laser-rangefinder). E) BL 60 Altimeter. F) UAV and Leica distometer and monitor display of the UAV camera at the canopy layer of the tree; the red circle indicates the tip area of the tree. When the tip is visible on the display monitor, the Leica distometer is shot to the drone its distance to the drone is measured as H1. The distometer is also used to measure the height above the ground level (H2). Height of tree = H1 + H2 (Adopted from Saliu *et al*., 2020 [1]).

For the thumb rule, the tree height was measured by stretching the arm out such that the top of the thumb aligned with the top of the tree and the base of the fist aligned with the base of the tree. While maintaining the same position, the observer rotated the thumb horizontally such that the base of the fist still aligned with the base of the tree. The tip of the thumb on the ground was marked and its distance from the base of the tree was measured as the tree height (Fig. 1A).

By following the stick method, the observer held a ruler while stretching out his arm and standing at a distance from the tree such that the top of the tree is aligned with the top of the ruler and the base of the tree is aligned with the hand holding the ruler (Fig. 1B). Once aligned, the distance from the hand grasping the base of the ruler to the observer's eye (= armlength), the distance from the hand to the top of the stick and, the distance from the observer to the base of the tree were all measured to estimate the tree height by following equation (Eq. 1) -

(1) $$Tree\;height=\frac{HS\;\times \;d}{AS\;}$$

where, *HS* is the length of stick from observer hand to its tip, *d* is the distance between observer and tree, and *AS* is the armlength.

The clinometer was operated by looking at the top of the tree with one eye and at the degree scale inside the device (angle α coinciding with tip of the tree) with the other eye simultaneously. The height of the observer's eye above the ground (*H2*) and the distance between the observer and the tree (*d*) were also recorded using a measuring tape. This method was carried out with the tree and the observer standing on the same ground level (Fig. 1C). The height of the tree was estimated by tangent method as shown below (Eq. 2) -

(2) TotaltreeheightH=H1+H2

H1=d×tan(α)

where, *H* is the total tree height, *d* is the distance between observer and tree (baseline distance), *α* is the angle correspond to treetop and *H2* is the distance from ground to the observer's eye.

The laser rangefinder possesses clinometer and rangefinder which allows both distance and height measurements. For height, the observer stood at a position from where the treetop and the base were clearly seen. The distance of the observer from the tree was either at 15, 20, 30 or 40 m. The mode of the device was set to “Hgt - Hgt2” which denotes the vertical separation between two points Hgt (treetop) and Hgt2 (tree base). The device was shot to the top and base of the tree (Fig. 1D) and the height of the tree was automatically computed and available to read from internal/external display of the device.

The altimeter measures tree heights by obtaining the elevation angle between the observer and the measured points on the tree using trigonometric principles. The observer stood at a known distance from the tree with a choice of 15, 20, 30 or 40 m. Each distance corresponds to a given scale on the device. The observer chose a distance at which the treetop and the base were clearly visible. The device has two white buttons (upper and lower) to control the movement of two needles on the measuring scale. The upper button was pressed when the device was shot at the treetop while the lower button was pressed when shot at the base of the tree. Holding these white buttons releases the corresponding needles and releasing the buttons fixes the needles. The height between the observer and the treetop/tree base can be read directly on the scale. The height measurement was done with the observer standing at the same ground level as the tree (Fig. 1E).

Each tree height measurement was made from a specific distance, either from 15 m, 20 m, 30 m or 40 m depending on the visibility of treetop and base, for the clinometer, laser rangefinder and altimeter. However, for thumb rule and stick method, the distance of observation went farther than 40 m (in the case of compartment 19A at the MMFR).

We flew the UAV from the base of a tree to the tip of the canopy vertically and recorded the altitude of above ground or sea level [2] displayed on-screen of a video tracking device (mobile phone: Huawei Nova 2 lite). The tree height was measured by pinpointing the UAV when it reached the top of the tree, visible from the UAV camera. This height was further confirmed by pointing a distometer to the base of the UAV (Fig. 1F). Saliu *et al.* (2020) discussed the implications of camera position, camera angle, tip recognition, wind and distometer accuracy on error in this approach.

Tree diameter at 130 cm ( D_130_ *sensu* Brokaw & Thompson [3]) above the ground or along the stem was measured for all trees using a diameter tape [4]. The angle of inclination was used as a proxy to measure the leaning nature of trees. This was done by placing a steel protractor at the base of the tree, and the angle at which the tree deviates from straightness (90°) was considered.

## CRediT Author Statement

**Ibrahim Sunkanmi Saliu:** Methodology, Data curation, Resources, Validation, Investigation,Funding acquisition, Visualization, Writing review & editing. **Giovanna Wolswijk:** Validation, Investigation, Visualization, Writing original draft, Writing review & editing. **Behara Satyanarayana:** Conceptualization, Methodology, Resources, Validation, Investigation, Supervision, Project administration, Funding acquisition, Writing review &editing. **Muhammad Amir Bin Fisol:** Investigation, Data curation, Writing review & editing. **Charles Decannière:** Validation, Supervision, Writing review & editing. **Richard Lucas:** Conceptualization, Methodology, Validation, Supervision, Project administration, Writing review & editing. **Viviana Otero:** Validation, Supervision, Project administration, Writing review & editing. **Farid Dahdouh-Guebas:** Conceptualization, Methodology, Resources, Validation, Investigation, Supervision, Project administration, Funding acquisition, Visualization, Writing review & editing

## Declaration of Competing Interest

The authors declare that they have no known competing financial interests or personal relationships which have, or could be perceived to have, influenced the work reported in this article.
